# MorTAL Kombat: the story of defense against TAL effectors through loss-of-susceptibility

**DOI:** 10.3389/fpls.2015.00535

**Published:** 2015-07-14

**Authors:** Mathilde Hutin, Alvaro L. Pérez-Quintero, Camilo Lopez, Boris Szurek

**Affiliations:** ^1^UMR IPME, Institut de Recherche Pour le Développement, IRD-CIRAD-Université Montpellier 2Montpellier, France; ^2^Biology Department, Universidad Nacional de ColombiaBogota, Colombia

**Keywords:** *Xanthomonas*, plant disease susceptibility *S* genes, hubs, TAL effectors, agricultural biotechnology, loss-of-function alleles

## Abstract

Many plant-pathogenic xanthomonads rely on Transcription Activator-Like (TAL) effectors to colonize their host. This particular family of type III effectors functions as specific plant transcription factors via a programmable DNA-binding domain. Upon binding to the promoters of plant disease susceptibility genes in a sequence-specific manner, the expression of these host genes is induced. However, plants have evolved specific strategies to counter the action of TAL effectors and confer resistance. One mechanism is to avoid the binding of TAL effectors by mutations of their DNA binding sites, resulting in resistance by loss-of-susceptibility. This article reviews our current knowledge of the susceptibility hubs targeted by *Xanthomonas* TAL effectors, possible evolutionary scenarios for plants to combat the pathogen with loss-of-function alleles, and how this knowledge can be used overall to develop new pathogen-informed breeding strategies and improve crop resistance.

## Introduction

*Xanthomonas* are a genus of plant pathogenic bacteria that cause devastating diseases on a wide range of hosts, leading to severe impact on yield quantity and quality of important crops such as rice, cassava, cotton, wheat, banana, mango, citrus, and cabbage (Schornack et al., 2013). In order to colonize their host, most of these pathogens rely on a type three secretion system (T3SS) specialized in the injection of virulence factors into the host cell. Also called type three effectors (T3Es), these proteins collectively promote the pathogen’s adaptation to specific host tissues, species, and genotypes. This adaptation includes suppression of plant immunity, nutrient acquisition, dispersal, or other virulence-related processes that benefit the pathogen. Upon translocation, T3Es localize to various subcellular compartments such as the plasma membrane or organelles.

The Transcription Activator-Like (TAL) effectors form a particular family of T3Es that act as bona fide plant transcription factors able to reprogram the host transcriptome following nuclear localization. They have a highly conserved structure that is modular and characterized by an N-terminal type three secretion signal, a C-terminal region bearing two to three nuclear localization signals and an acidic transcription activation domain. In between is a central region built of quasi-identical tandem direct repeats of 33–35 amino-acids forming a unique type of DNA-binding domain. Each repeat folds into a super-helical structure that wraps the DNA. Each repeat forms a two-helix bundle in which so-called repeat variable di-residues (RVDs) at positions 12 and 13 reside on the interhelical loop that projects into the DNA major groove. More precisely, RVDs mediate the binding of TAL effectors to the double-strand DNA in a sequence-specific manner with residue 12 stabilizing the overall interaction while residue 13 specifically contacts the DNA base (reviewed in Mak et al., 2013). Functional differences between TAL effectors are therefore mainly due to the nature of the string of RVDs that determine the sequence of the so-called TAL effector binding element (EBE) in host promoters. TAL effectors can be found in most *Xanthomonas* species and related proteins are also present in *Ralstonia solanacearum* (RipTALs; de Lange et al., 2013) and *Burkholderia rhizoxinica* (Bats; Juillerat et al., 2014; Lange et al., 2014).

Several *Xanthomonas* TAL effectors are known to play an essential role during infection, i.e., the deletion of the genes encoding them leads to reduced disease. Plant genes may contribute to disease susceptibility in different ways, notably by favoring pathogen attraction and attachment to the host, controlling the establishment of a favorable environment for host tissue penetration, colonization, or pathogen dispersal (reviewed by Lapin and Van den Ackerveken, 2013). In the case of plant pathogenic bacteria our knowledge on the diversity and function of disease determinants is still scarce. However, recent breakthroughs in the field of TAL effector biology are significantly improving our understanding of bacterial disease processes, thereby fostering innovative disease control strategies. In this review, we analyze host–pathogen interactions over evolutionary time using the popular video game Mortal Kombat^1^, in which combatants strike and counter to survive an epic battle, as a metaphor. We initially focus on the mechanisms underlying susceptibility caused by TAL effectors. Then we explore the strategies that plants have evolved to counter the action of TAL effectors, with emphasis on the strategy of “resistance through loss-of-susceptibility” achieved by avoiding the binding of TAL effectors to host DNA using mutations of their binding sites. Finally, we discuss how, in light of these findings, natural or engineered resistance can be bred to control TAL effector-mediated diseases.

## Round 1: Bacteria Attack Using TAL Effectors to Induce Susceptibility Genes

Transcription Activator-Like effectors are found in many plant pathogenic *Xanthomonas* species where they significantly contribute to disease development but their exact function as minor or major virulence factors has been disentangled for only a few of them (Boch and Bonas, 2010). The number of *TAL* effectors genes contained in the genome of a *Xanthomonas* strain is highly variable between strains. For example, strains of *Xanthomonas oryzae* pv. oryzae (*Xoo*) contain from 9 up to more than 15 different TAL effectors. Yet it has been shown that only one or two of them play an essential role in pathogenicity and encode major virulence factors (Yang and White, 2004). For these strains the mutation of the corresponding genes leads to no development of the disease (Yang and White, 2004). The genes targeted by these TAL effectors are called (*S*) susceptibility genes because their induction is essential to the complete development of the disease. Unlike resistance (*R)* genes that are usually expressed only in the presence of the pathogen, most of the known *S* genes play roles in plant development, and are exploited by pathogens through their overexpression during the infection. The discovery of the TAL effector-DNA binding code combined with transcriptomic data has led to the identification of a *S* gene list for different *Xanthomonas*/host interactions (**Table 1**). Notably, many of these *S* genes encode either transporters (sugar or sulfate) or transcription factors, and their induction is hypothesized to facilitate bacterial colonization and symptom development.

**Table 1:** Plant susceptibility or candidate *S* genes targeted by *Xanthomonas* Transcription Activator-Like (TAL) effectors.

Target function	Target name	TAL name	Specie	Host	Symptoms	Effect	Reference	Loss *S* alleles	Reference
Transcription factor	*UPA20*	AvrBs3	*Xav*	Pepper	Cell hypertrophy	Increased egress to the leaf surface	Kay et al. (2007)		
	*CsLOB1*	PthA series	*Xcc*	Citrus	Cell expansion	Canker	Hu et al. (2014), Li et al. (2014)		
		PthB	*Xca*	Citrus	Cell expansion	Canker	Al-Saadi et al. (2007), Hu et al. (2014)		
		PthC	*Xca*	Citrus	Cell expansion	Canker			
	*TFX1*	PthXo6	*Xoo*	Rice	Water soaking	Increased growth and lesions	Sugio et al. (2007)		
	*TFIIAγI*	PthXo7	*Xoo*	Rice	Water soaking	Increased growth and lesions			
Sugar Transporter	*CsSWEET1*	PthA series	*Xcc*	Citrus	Unknown	Unknown	Hu et al. (2014)		
	*OsSWEET11*	PthXo1	*Xoo*	Rice	Water soaking	Increased growth and lesions	Chu et al. (2006), Yang et al. (2006)	*xa13*	Chu et al. (2006), Yang et al. (2006)
	*OsSWEET13*	PthXo2	*Xoo*	Rice	Water soaking	Increased growth and lesions	Zhou et al. (2015)	*xa25*	Liu et al. (2011), Zhou et al. (2015)
	*OsSWEET14*	PthXo3	*Xoo*	Rice	Water soaking	Increased growth and lesions	Yang and White (2004)	TALEN-based disruption	Li et al. (2012)
		AvrXa7	*Xoo*	Rice	Water soaking	Increased growth and lesions	Antony et al. (2010)		
		Tal5	*Xoo*	Rice	Water soaking	Increased growth and lesions	Streubel et al. (2013)		
		TalC	*Xoo*	Rice	Water soaking	Increased growth and lesions	Yu et al. (2011)		
	*MeSWEET10a*	TAL20	*Xam*	Cassava		Unknown	Cohn et al. (2014)		
Sulfate transporter	*SULTR3;6*	Tal2g	*Xoc*	Rice	Water soaking	Increased lesions and egress to the leaf surface	Cernadas et al. (2014)		
miRNA stability	*OsHEN1*	PthXo8	*Xoo*	Rice		Not reported	Moscou and Bogdanove (2009)		
		Tal1C	*Xoc*	Rice		None detected			

*Xcv, Xanthomonas axonopodis pv. vesicatoria; Xcc, X. citri pv. citri; Xca, X. citri pv. aurantifolii; Xoo, X. oryzae pv. oryzae; Xoc, X. oryzae pv. oryzicola; Xam, X. axonopodis pv. manihotis*.

The SWEET family of sugar transporters represents the best characterized group of *S* genes induced by TAL effectors (Chen, 2014). In rice, these include *OsSWEET11 (a.k.a Xa13/xa13)* activated by PthXo1 from strain *Xoo* PXO99^A^, *OsSWEET13 (a.k.a Xa25/xa25)* activated by PthXo2 from *Xoo* strains JXO1^A^ and MAFF311018, and *OsSWEET14* activated by AvrXa7, PthXo3, TalC, and Tal5 from *Xoo* strains PXO86, PXO61, BAI3, and MAI1, respectively (Yang and White, 2004; Chu et al., 2006; Antony et al., 2010; Yu et al., 2011; Streubel et al., 2013; Zhou et al., 2015). They are all members of clade III of the *SWEET* gene family. It was shown that in rice, all five members of clade III can act as a major *S* gene (Li et al., 2013; Streubel et al., 2013). Interestingly, related genes are also induced by *Xanthomonas* TAL effectors in other hosts. For instance *MeSWEET10a* and *CsSWEET1* are, respectively, induced by TAL20 from *X. axonopodis* pv. manihotis *(Xam)* in cassava and the PthA series from *X. citri* ssp. *citri* in citrus (Cohn et al., 2014; Hu et al., 2014). *Xam*-mediated induction of *MeSWEET10a* which also belongs to clade III is essential for full development of watersoaking as infiltration of strain Xam668Δ*TAL20* leads to reduced symptoms (Cohn et al., 2014). Intriguingly the induction of *MeSWEET10a* is necessary for symptom expansion but not for bacterial growth. In contrast, *CsSWEET1* belongs to clade I and no function for citrus canker has been demonstrated to date (Hu et al., 2014). Similarly, AvrBs3 from *X. axonopodis* pv. vesicatoria induces *UPA16* from pepper which also belongs to the *SWEET* family but there is no evidence for this gene to play a role for bacterial spot of pepper and tomato (Kay et al., 2009).

At least two members of the *SWEET* family in rice were shown to play a crucial role in plant development. Homozygous lines containing a T-DNA insertion in *OsSWEET14* display delayed growth and seeds smaller than the wild type plant (Antony et al., 2010), and plants silenced for *OsSWEET11* have reduced fertility (Yang et al., 2006). They encode sugar transporters that allow the eﬄux of carbohydrates from the cell to the intercellular space. Thus, their induction could make carbohydrates easily available for bacteria (Chen et al., 2012), though to date it has not been clearly demonstrated that these carbohydrates help bacterial growth directly. *SWEET* genes may play a role in multiple plant pathogen interactions (Lemoine et al., 2013). *VvSWEET4* in grapevine is up regulated during the interaction with the gray mold fungus *Botrytis cinerea* and a knockout mutant in *Arabidopsis* for the orthologous gene *AtSWEET4* becomes less susceptible to this pathogen (Chong et al., 2014). Also in *Arabidopsis*, differential expression of *SWEET* genes is observed after infection by *Pseudomonas syringae* pv. tomato, *Golovinomyces cichoracearum*, and *Plamodiophora brassicae* (Siemens et al., 2006; Chen et al., 2010) suggesting that numerous pathogens target the sugar transport mechanisms of the host upon infection.

While *SWEET* genes are essential for the vascular pathogen *Xoo* to cause Bacterial Blight in rice, they are curiously not targeted by the closely related non-vascular pathovar *X. oryzae* pv. oryzicola (*Xoc*) that is responsible for bacterial leaf streak of rice. It is then tempting to speculate that induction of *S* genes by TAL effectors may explain tissue specificity to some extent. However, transformation of *Xoc* with *OsSWEET-*inducing TAL effectors from *Xoo* does not allow vascular colonization by *Xoc* (Verdier et al., 2012). Nevertheless, the role of TAL effectors in defining host-ranges and colonization mechanisms is still a field worth exploring. Recently, the systematic mutagenesis of each *TAL* effector gene of *Xoc* strain BLS256 completed with the functional analysis of their potential targets unmasked a new *S* gene specifically required for leaf streak. *OsSULTR3;6* encodes a sulfate transporter targeted by Tal2g and its induction is necessary for lesion expansion and bacterial exudation but not for bacterial growth (Cernadas et al., 2014). How this sulfate transporter contributes to disease has yet to be elucidated. One hypothesis is that its induction modifies the redox potential in a favorable manner for symptom establishment and bacterial expansion. Some orthologous genes have been identified to play a similar role in other pathosystems and in establishment of symbiosis (Cernadas et al., 2014).

Another group of functionally related genes targeted by TAL effectors are transcription factors. The first identified was *UPA20* in pepper. This transcription factor induced by AvrBs3 from *X. axonopodis* pv. vesicatoria belongs to the *bHLH* family and trans-activates the secondary target *UPA7* (Kay et al., 2007). The expression of *UPA7* leads to enlargement of mesophyll cells of pepper leaves that could promote propagation of bacteria (Kay et al., 2007). Another transcription factor acting as a major susceptibility gene is *CsLOB1*, required for pustule formation during citrus bacterial canker. This gene is targeted by five different TAL effectors, including PthA4, PthA^w^, and PthA^∗^ from *X. citri* ssp. *citri* and PthB and PthC from *X. citri* ssp. *aurantifolii* (Hu et al., 2014). Interestingly an *Arabidopsis* protein belonging to the LOB family is described as a susceptibility gene for the fungal pathogen *Fusarium oxysporum* (Thatcher et al., 2009). The specific functions in development of CsLOB1 are not known but it was shown that proteins with LOB domain are responsive to numerous phytohormones, play a role in anthocyanin and nitrogen metabolism and are involved in the regulation of lateral organ development (Majer and Hochholdinger, 2011; Gendron et al., 2012). *OsTFXI* a bZIP transcription factor and *OsTFIIAγ1* a general transcription factor, are targeted, respectively, by PthXo6 and PthXo7 from *Xoo* strain PXO99^A^ (Sugio et al., 2007). *OsTFXI* is particularly interesting as it is induced by all *Xoo* strains assessed to date (White and Yang, 2009). In contrast to the wild type, a transgenic plant overexpressing *OsTFXI* is fully susceptible to a *pthXo6* mutant confirming that it is an *S* gene (Sugio et al., 2007). How *OsTFXI* promotes disease is not yet understood.

**FIGURE 1:**
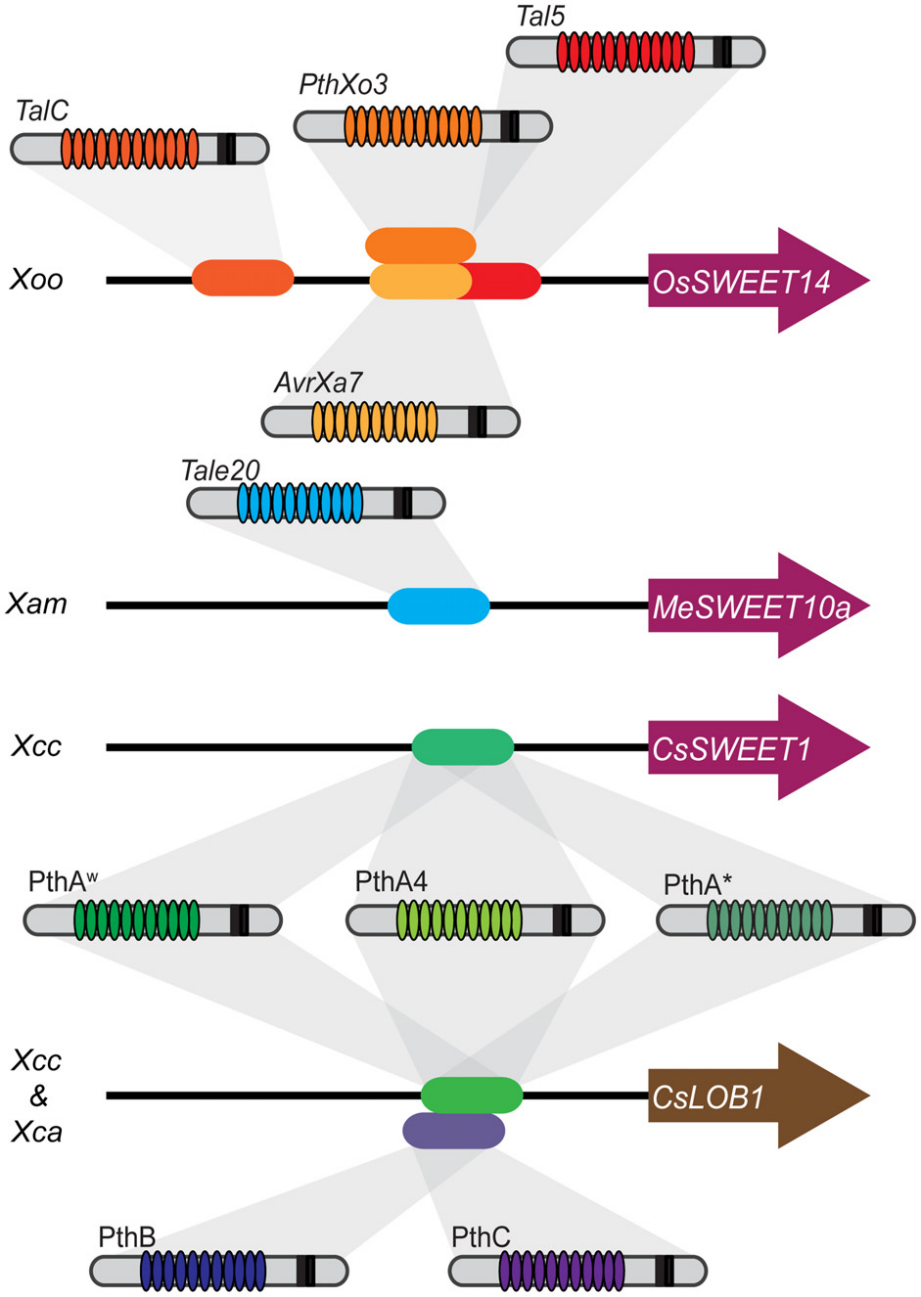
**Functional convergence in bacterial Transcription Activator-Like (TAL) effectors and plant targets.** Several susceptibility targets of *Xanthomonas oryzae* pv. oryzae belong to Clade III of the *SWEET* family. Four TAL effectors from diverse *Xoo* strains belonging to different lineages and from distinct geographical origins target *OsSWEET14*. TalC and Tal5 from the two *Xoo* African strains BAI3 (Burkina Faso) and MAI1 (Mali) bind to two distinct effector binding elements (EBEs). In addition, the *OsSWEET14* promoter is targeted by two *Xoo* Philippine strains, PXO86 and PXO61 through the TAL effectors AvrXa7 and PthXo3. The corresponding EBEs partially overlap with the Tal5 EBE. The repeat variable di-residue (RVD) arrays and the number of repeats (not represented in the figure) for these three TAL effectors are significantly different, highlighting evolutionary convergence. Not shown in the figure is the case of the resistance gene *Xa7* that recognizes AvrXa7 but not PthXo3 that is slightly divergent from AvrXa7. A similar case of convergence is observed for the induction of *CsLOB1* which is targeted by PthB and PthC from *X. citri* ssp. *aurantifolii* and the PthA series from *X. citri* ssp. *citri*. PthA4, PthA^w^, and PthA^∗^ (but not PthB and PthC) induce *CsSWEET1* belonging to the Clade I of the *SWEET* family. However, a role in disease development has not been shown. A third species, *X. axonopodis* pv. *manihotis*, targets through TAL20 a member of the *SWEET* family, *MeSWEET10a*, that belongs to Clade III and acts as a susceptibility gene.

In summary, for *Xoo* species, the SWEET transporters are a major target in rice and among them *OsSWEET14* is targeted by four different TALs from genetically and geographically distant *Xoo* strains. The *SWEET* genes are targeted by at least three other *Xanthomonas* species in three other hosts. There is undoubtedly an evolutionary convergence for the induction of this family inter species and intra pathovar. This convergence occurs also for the transcription factors. Five different TAL effectors with different RVD content are able to induce *CsLOB1*, which seems to be the single susceptibility gene induced by both *X. citri* pathovars for the establishment of citrus canker (Hu et al., 2014). Thus, convergence seems to be a good indicator for the discovery of *S genes* (**Figure 1**). A similar behavior of convergence toward a selected group of targets in the host has also been found for non-TAL effectors from different pathogen groups in *Arabidopsis* (Mukhtar et al., 2011). In addition, computational predictions suggest that targeting of functional “hubs” is indeed a common feature for TAL effectors (Perez-Quintero et al., 2013).

Taking this into account, new potential *S* genes arise, including for example *OsHEN1* in rice. *OsHEN1* encodes a methyl transferase and is the only common target of *Xoo* and *Xoc* via, respectively, PthXo8 and Tal1c (Moscou and Bogdanove, 2009). Although a *tal1c* mutant showed no reduction in virulence (Cernadas et al., 2014), and data for PthXo8 are yet to be reported, the convergence of different pathovars onto this target may suggest an important role in the respective diseases. *OsHEN1* plays important roles in different physiological processes through the stabilization of small RNAs, introducing the exciting question of how induction of this gene aids in bacterial colonization. On the other hand, there are some TAL effector for which a role in pathogenicity has been shown but the target remains unknown or unconfirmed (Yang et al., 1994; Castiblanco et al., 2012; Munoz-Bodnar et al., 2014).

## Round 2: Plant Dodges Bacteria’s TAL Effectors Through Mutation in Promoters of *S* Genes

The evolutionary convergence of TAL effector activity on a restricted set of key host genes of physiological importance (**Figure 1**) implies that some allelic variants (those that can avoid binding on the effector) could confer broad-spectrum resistance to *Xanthomonas.* To date, EBE variants that abolish TAL effector binding have been found in the promoter of two major susceptibility genes: *OsSWEET11* and *OsSWEET13*, resulting in the recessive resistance alleles known, respectively, as *xa13* and *xa25* (Chu et al., 2006; Yang et al., 2006; Liu et al., 2011; Zhou et al., 2015).

The promoter of the susceptible allele of *OsSWEET11:Xa13* but not of *xa13* is induced directly by the TAL effector PthXo1 from PXO99^A^ (Yang et al., 2006; Romer et al., 2010). Varieties containing *xa13* are resistant to PXO99^A^ and the recessive resistance observed is not characterized by the typical hypersensitive response phenotype as it results only on the loss of the induction of a gene essential to disease development (Yuan et al., 2009, 2011). There is a collection of rice varieties naturally presenting an *xa13* allele. All these alleles differ from the *Xa13* genotype by insertion, deletion, or substitution in the PthXo1 EBE (Romer et al., 2010). Interestingly a single substitution in the second nucleotide of the EBE is sufficient to avoid induction of *OsSWEET11* by PthXo1. In the case of *OsSWEET13*, the susceptible allele *Xa25* but not the race specific recessive resistance gene *xa25* is induced by the *Xoo* strain PXO339 (Liu et al., 2011). Transformation of *Xa25* in a resistant variety with a *xa25* genotype led to susceptibility to PXO339 (Liu et al., 2011). Recently it was shown that PthXo2 directly induces *Xa25*. *Xa25* differs from *xa25* by a one nucleotide deletion in the EBE of PthXo2. Promoter mutation generated by CRISPR/Cas9 technology confirmed that *OsSWEET13* induction is essential for strains that depend on PthXo2 to lead to disease susceptibility (Zhou et al., 2015).

For *OsSWEET14*, no naturally occurring resistance alleles have been reported yet, but they have been genetically engineered. Li et al. (2012) generated loss of susceptibility alleles altered in the box targeted by AvrXa7 and PthXo3 using the TALEN strategy, an approach based on TAL effector binding domains coupled with nucleases (reviewed in Bogdanove and Voytas, 2011; Carroll, 2014). They showed that a deletion of 4 bp is sufficient for loss-of-susceptibility to AvrXa7 and PthXo3. As discussed below Round 4), this genome editing strategy could be extensively used to develop resistant varieties to *Xanthomonas* in various systems.

It is expected that more naturally occurring loss-of-susceptibility alleles can be found by promoter screening of *S* genes in different host germplasm collections (see Round 4). An example of a resource for this type of screening is the recently released SNP data from rice obtained from the sequencing of the genomes of 3000 rice varieties, available in the SNPseek database (Alexandrov et al., 2015). Surprisingly when using this data to look for SNPs in a set of known EBEs in rice (not all from *S* genes) very few SNPs are found within the EBEs (**Table 2**). It is worth noting, that no indels are included in the SNPseek data and that all the SNPs were obtained from mapping NGS reads onto only one reference genome (Alexandrov et al., 2015). So it is possible that more SNPs in these regions will be found using different tools and datasets.

**Table 2:** SNPs found in 3000 rice genomes at the EBEs of known TAL effector targets in rice.

TAL	Xo Strain	Reference	Gene	Effector binding element (EBE) sequence	SNPs EBE	SNPs promoter	SNPs gene
PthXo3	PXO61	Yang and White (2004)	LOC_Os11g31190 (SWEET14)	TATATAAACCCC**C**TCCAACCAGGTG**C**TAAG	2	20	28
AvrXa7	PXO86	Antony et al. (2010)	LOC_Os11g31190 (SWEET14)	TATATAAACCCC**C**TCCAACCAGGTG**C**T	2	20	28
Tal3b	BLS256	Cernadas et al. (2014)	LOC_Os07g36430	TATAAGAAGCCCTC**A**CTCG	1	22	3
Tal9b	BLS256		LOC_Os01g51040	TAGAAAC**T**GCTCTTCCT	1	2	22
TalC	BAI3	Yu et al. (2011)	LOC_Os11g31190 (SWEET14)	CATGCATGTCAGCAGCTGGTCAT	0	20	28
Tal5	MAI1	Streubel et al. (2013)	LOC_Os11g31190 (SWEET14)	TAAGCTCATCAAGCCTTCA	0	20	28
PthXo6	PXO99A	Sugio et al. (2007)	LOC_Os09g29820 (OsTFX1)	TATAAAAGGCCCTCACCAACCCAT	0	5	3
PthXo7	PXO99A		LOC_Os01g73890 (OsTFIIAγ1)	TATAATCCCCAAATCCCCTCCTC	0	8	8
PthXo1	PXO99A	Yang et al. (2006)	LOC_Os08g42350 (SWEET11)	TGCATCTCCCCCTACTGTACACCAC	0	8	17
Tal9A	PXO99A	Moscou and Bogdanove (2009)	LOC_Os07g06970 (HEN1)	TCCCTTCCCTAAACCCCACTT	0	4	20
Tal1c	BLS256	Moscou and Bogdanove (2009)	LOC_Os07g06970 (HEN1)	TCCCCCTCGCTTCCCTT	0	4	20
Tal2c	BLS256	Cernadas et al. (2014)	LOC_Os03g03034	TCCGGCCCCTCTCCCCCCGCCACCTGAC	0	5	25
Tal2d	BLS256		LOC_Os04g49194	TATTCCCTCGCGTGATC	0	18	78
Tal3b	BLS256		LOC_Os02g34970	TATAAGTAGCACTCGCTCA	0	4	9
Tal3b	BLS256		LOC_Os05g27590	TATAAATAGCCCTCACTCT	0	3	0
Tal4a	BLS256		LOC_Os03g37840	TATATATCTCGGTCAGGCCAGGCCCCT	0	12	28
Tal3c	BLS256		LOC_Os03g07540	TACACGTTCCCTCCACC	0	3	9
Tal3c	BLS256		LOC_Os02g47660	TACACATTAGCTACCAT	0	2	11
Tal6	BLS256		LOC_Os09g29100	TACAAAGAGAACGCATCCCCC	0	11	24
Tal6	BLS256		LOC_Os12g42970	TAAAAGCGAAACCCTCCCTCC	0	16	8
Tal5a	BLS256		LOC_Os02g15290	TAAATCCCCCCTGCACAGGAAAGCT	0	31	2
Tal2g	BLS256		LOC_Os01g52130	TGGCCCGTAGCCTCTCCT	0	0	89
Tal2g	BLS256		LOC_Os06g46500	TGGCAAGTGACCTCAGCT	0	9	14
Tal4c	BLS256		LOC_Os06g37080	TATAAAACCTGGACAAGCCTCTCT	0	7	46
Tal4b	BLS256		LOC_Os09g32100	TGTATAGTATCCCCT	0	14	14

*SNPs found elsewhere in the promoter regions (1 kb upstream TSS) and the genes (including intrond) are also indicated. Position of the SNPs in the EBE sequences is indicated by bold and underlined nucleotides. SNP data were obtained from the Rice SNPseek database (Alexandrov et al., 2015)*.

Nonetheless, this raises the question: **how common are loss-of-susceptibility alleles of *S* genes?**

Gaining resistance through loss-of-susceptibility alleles requires the plant to mutate a region of the promoter to avoid binding of the TAL effector without altering physiologically important regulatory elements in the promoter. This might be particularly hard to achieve given that many TAL effectors seem to bind to crucial elements of the promoter. As a matter of fact, most of the known functional binding sites for TAL effectors are located in a region overlapping with that of core promoter elements of the gene (between 300 bp upstream and 200 bp downstream of the transcription start site). Indeed, TAL effectors seem to bind predominantly to functional motifs, such as the TATA box and the TC box (Grau et al., 2013; Pereira et al., 2014).

Regulatory elements in plant promoters are usually very short; median observed length of 8 bp and notoriously conserved (Reineke et al., 2011). Point mutations (transversions and transitions) in these elements may prevent binding of endogenous plant TFs and probably will not be selected unless they offer a great improvement in fitness (i.e, resistance to pathogenic bacteria). However, point mutations in an EBE could fail to prevent binding since the way TAL effectors bind to DNA allows for enough flexibility to tolerate “mismatches”. For instance some RVDs are able to indiscriminately accommodate different nucleotides, particularly those RVDs with weaker interactions with DNA (Streubel et al., 2012). Also, RVDs in the C-terminal region of TAL effectors seem to be particularly tolerant of mismatches (Meckler et al., 2013; Perez-Quintero et al., 2013). So a theoretical scenario for point mutations in an EBE to be effectively selected would be if they occur in a position normally bound by a strong-effect RVD in the N-terminal region. This seems to be the case of the *xa13* alleles found in the rice varieties Tepa1, BJ1, Chinsurah 11484, Chinsurah 11760, and Chinsurah 50930 (Romer et al., 2010) where a single nucleotide substitution in the position matching the first RVD of PthXo1 is enough to prevent binding of the TAL effector. It would be interesting to test whether the SNPs found in the EBEs in **Table 2** can prevent the binding of the corresponding TAL effector.

An alternative route to produce loss-of-susceptibility alleles is through deletion and insertion. Since *cis*-elements are often redundant, modular, and can exert their function regardless of their order in the promoter (Vedel and Scotti, 2011), indels and recombination events could in theory occur in some areas in the promoter between *cis-*elements or in areas including redundant elements without affecting gene regulation. These events could be more effective in defeating binding of TAL effectors since, with some exceptions (discussed under Round 3, below), TAL effectors do not tolerate gaps in their binding site as shown in numerous assays (Boch et al., 2009; Streubel et al., 2012; Richter et al., 2014). Indeed, many of the resistant *xa13* alleles found in various rice varieties contain insertions or deletions in the promoter that disrupts the EBE found in susceptible varieties (Chu et al., 2006; Yang et al., 2006; Romer et al., 2010) and the resistant allele of *xa25* is caused by a one-nucleotide deletion in the promoter in resistant varieties (Zhou et al., 2015). It is possible that indels in the promoters of *S* genes are a more common way of generating resistance, which would explain why such low variability is found in the SNPseek data (**Table 2**) that does not consider indels.

It is worth noting that there are other ways for the plant to defend itself against TAL effectors than through loss-of-susceptibility alleles, notably by using the TAL effector machinery against bacteria and “tricking” them into inducing resistance genes: the mechanism of the so-called executor genes is reviewed elsewhere (Boch et al., 2014). Additionally, plants can also directly recognize TAL effectors using “traditional” resistance genes, as is the case of *Bs4* from tomato, encoding a NBS-LRR protein that directly recognizes the TAL effectors AvrBs4, Hax3, and Hax4 and subsequently activates defenses (Schornack et al., 2004; Kay et al., 2005).

## Round 3: Bacteria Strike Back With New Weapons

On the bacterial side, resistance through loss-of-susceptibility could be overcome in a couple of ways. On one hand, it is possible that bacteria could overcome the effect of mismatches between TAL effectors and their cognate EBEs. For example, Richter et al. (2014) showed that some TAL effectors contain aberrant-length repeats that can “loop-out” when the TAL effector binds to the EBE thus allowing the recognition of target DNA sequences with a -1 nucleotide frame-shift, which allows for more flexibility in binding.

PthXo3 contains an aberrant repeat that apparently helps it to bind to the promoter of *OsSWEET14* by disengaging from the interaction to shift the interaction register downstream. AvrXa7 also has an aberrant repeat that might function the same way at *OsSWEET14*, though in this case EBEs are predicted both with and without the register shift (Richter et al., 2014). Furthermore, by using artificial TAL effectors it was shown that indeed a TAL effector containing an aberrant repeat could target both the “susceptible” and “resistant” version of the promoter of the susceptibility gene *Xa25* (*OsSWEET13*; Richter et al., 2014).

Another way for bacteria to counteract loss-of-susceptibility is by acquiring and harboring functionally redundant TAL effectors. Different TAL effectors could target dissimilar EBEs or variants of the promoter susceptibility gene. Alternatively several TAL effectors can activate redundant susceptibility genes. One example of the latter can be found in the *Xoo* strain MAFF311018 that contains AvrXa7 that can induce *OsSWEET14*, as well as PthXo2 that induce the susceptible allele of *OsSWEET13* (Perez-Quintero et al., 2013; Zhou et al., 2015). TAL effectors could also diverge to avoid recognition by resistance genes as exemplified by the case of AvrXa7 and PthXo3, where, despite both effectors binding to overlapping EBEs in the *OsSWEET14* promoter (Antony et al., 2010), PthXo3 presumably avoids *Xa7*-triggered resistance (White and Yang, 2009).

**FIGURE 2:**
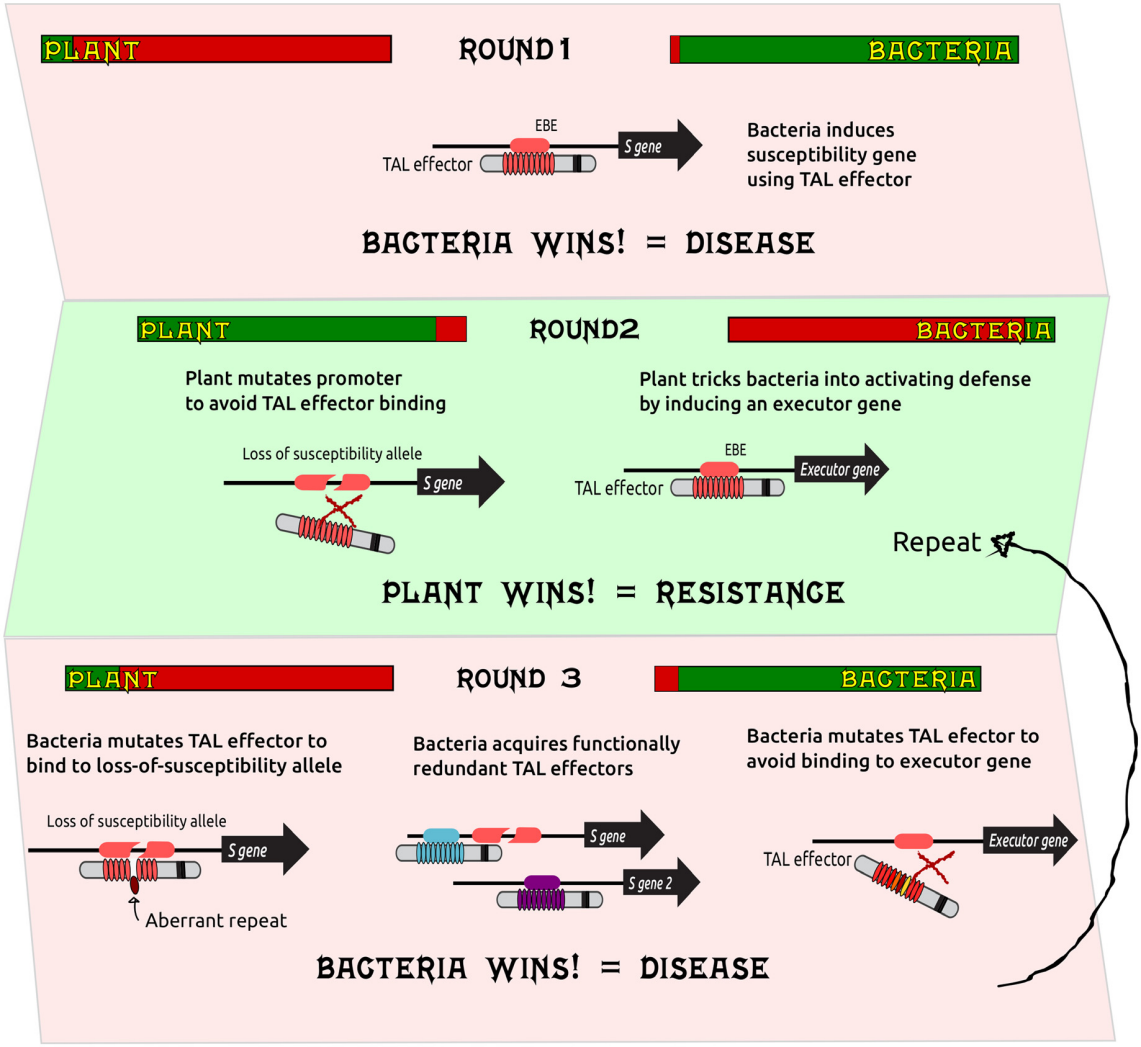
**A simplified model for combat involving bacterial TAL effectors and plant susceptibility genes.** To cause disease, bacteria use TAL effectors that bind to the promoter regions of susceptibility (*S*) genes. The plant might defend itself against these effectors through mutations in the promoter that prevent binding of the TAL effector(s): so called loss-of-susceptibility alleles. Alternatively, plants can trick bacteria into inducing defenses via an executor gene. Bacteria can in turn counter by mutating TAL effectors to recognize the loss-of-susceptibility alleles using for example aberrant repeats that can accommodate single base deletions in the EBE or by mutating TAL effectors to avoid binding to the promoters of executor genes. Bacteria can also acquire new TAL effectors to redundantly induce *S* genes, the plant can evolve new loss-of-susceptibility alleles and executor genes, and the process becomes cyclical. Bars for plant and bacterium represent “health bars” typically used in video games to indicate the extent to which a combatant is winning or losing. Bold black arrows represent genes.

We have proposed a simplified model for the pathogen-host combat (**Figure 2**) where, in response to disease caused by the induction of *S* genes by TAL effectors (Round 1), plants evolve loss-of-susceptibility alleles predominantly through the selection of mutations that impede binding of the TAL effectors to their cognate EBEs but that ideally do not disrupt plant transcription factor binding sites. Alternatively, plants can trick bacteria into activating defenses by acquiring EBEs for bacterial TAL effectors in the promoter regions of resistance genes, the so-called executor genes (Round 2). It is also possible that executor genes arise from susceptibility genes that become resistance genes through selection. In response, bacteria can mutate TAL effectors to recognize the loss-of-susceptibility alleles (for example, by acquiring aberrant repeats) as well as mutate TAL effectors to avoid the induction of executor genes, or they can incorporate an arsenal of functionally redundant TAL effectors giving bacteria multiple ways to induce *S* genes, or to induce functionally equivalent *S* gene paralogs.

Of course, for each new *S* gene induced by bacteria, new selection pressure arises for the plant to avoid binding, and the rounds can repeat themselves indefinitely. Maybe the large repertoires of TAL effectors found in some strains are constituted by fallen players in the combat beaten by the plants defenses, and/or by tag team players, waiting to get into the ring and overkill (i.e., strike a fatality).

## Round 4: Researchers Enter the Ring

**FIGURE 3:**
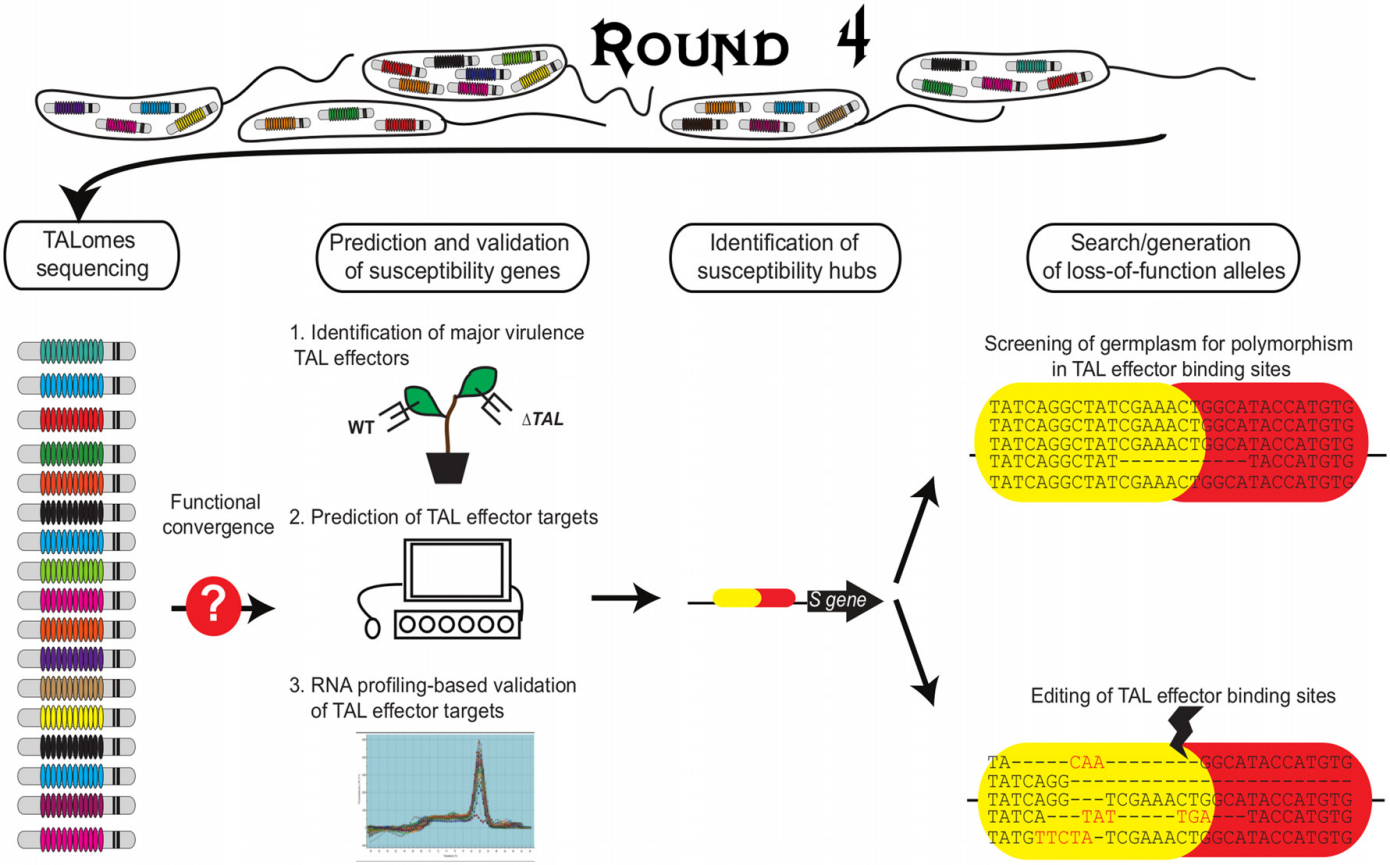
**Exploiting TAL effector knowledge to identify *S* genes and loss-of-function alleles.** Based on diversity studies conducted on a particular *Xanthomonas* species, it is possible to direct the research for virulence targets from the most prevalent TAL effectors. Field surveys allow the collection of strains at a regional or national scale. All or representative ones are selected to characterize the TALomes, i.e., their complete repertory of TAL effectors. The corresponding *S* genes can be identified employing three complementary strategies. First, the expected major virulence role of a prevalent TAL effector has to be assessed *in planta* (by loss of function mutational analysis or heterologous expression for example). Second, different bioinformatics algorithms (TALVEZ, TAL Effector Nucleotide Targeter, Talgetter) based on the TAL-DNA binding specificity code are employed to predict TAL effector targets in the host genome (assuming that at least a reference genome is available). Finally, RNA profiling strategies are employed to identify TAL effector-dependent differentially expressed genes. Any such genes with a predicted EBE for the TAL effector are strong candidates. For these, functional characterization using designer TAL effectors is the next step (Boch et al., 2014). Then, bioinformatics and functional analyses can be used to identify *S* hubs. The identification of natural variants in the EBEs from germplasm databases or Ecotilling or the generation of EBE mutations by genome editing in specific EBEs will lead to loss-of-function alleles as new plant disease resistance sources.

Genetic engineering strategies to improve disease resistance are based on the knowledge of the mechanism employed by plants to recognize pathogen proteins. Although resistance to virus and to some extent *Xanthomonas* is mediated by recessive genes, efforts mainly focused on the identification of dominant *R* genes and the transformation of susceptible varieties using these genes. This type of resistance is often strain-specific and can be easily overcome by the pathogen through mutations in unique and specific avirulence genes (Dangl et al., 2013). In contrast, the knowledge of TAL effector mechanisms offers new exciting opportunities for researchers aiming to develop resistant varieties. A research strategy that exploits TAL effector knowledge to generate broad-spectrum and durable resistance (**Figure 3**) might include:

### The Identification of Major TAL Effectors in Pathogen Populations of Interest

The characterization of a complete or near-complete TAL effector repertoire for a large group of strains of a particular *Xanthomonas* species, collected in geographically diverse regions and through different time periods, allows for the establishment of the more prevalent TAL effectors for that population (**Figure 3**). The sequencing of TAL effectors is a very complicated task given their repetitive nature. However, through the employment of new, relatively low cost, long-read sequencing technologies such as single molecule, real-time sequencing (Pacific Biosciences), it is possible to accurately assemble complete TAL effector sequences from whole genome data (A. Bogdanove, personal communication). A more difficult and time-consuming strategy is to clone and sequence TAL effectors individually. A non-tested way could be to generate TAL effector-specific libraries and sequence them. The knowledge gained on “TALome” diversity will allow estimation of the durability of a loss-of-susceptibility allele or an executor gene, which can be anticipated depending on the conservation of a particular TAL effector or a group of functionally equivalent TAL effectors in a given population. Also, a prevalent TAL effector can be taken as candidate virulence factor and prioritized for functional characterization. These TAL effectors can then be tested experimentally to confirm whether or not they have a role in virulence. This knowledge about TALome diversity will be key to design the best breeding resistance strategy. The next step would be:

### The Prediction and Validation of Susceptibility Hubs in the Host

Any host gene representing a benefit to bacteria is a potential plant susceptibility gene and can be considered as a candidate for transformation strategies. However, considering that the aim is to provide broad and durable resistance, those *S* genes that constitute convergence hubs for various TAL effectors (like the SWEET family) are ideal. These could be identified by combining EBE prediction using the RVD sequences for the major virulence TAL effectors identified and expression data (RNA-seq or microarray). Functional characterization of the *S* gene would also be ideal.

Once a key *S* gene targeted by one or more major TAL effectors is identified, a logical step is:

### The Screening for Natural Loss-of-Susceptibility Alleles in the Germplasm

For this, researchers can take advantage of the new sequencing technologies and the public genome sequence databases. In addition to the Rice SNPSeek data (Alexandrov et al., 2015), and other genomic databases for rice (Yonemaru et al., 2014; Zhao et al., 2015), similarly large datasets exist for other hosts worth exploring. In *Arabidopsis*, the 1001 project as of September 2014 includes the sequencing of over 1000 lines, representing a valuable resource to find resistance sources for *X. campestris* (Weigel and Mott, 2009). In cassava, an important resource consisting of the partial genome for 600 accessions is available and can be exploited to identify polymorphism in EBEs for targets of TAL effectors from *X. axonopodis* pv. manihotis^2^. In tomato the sequence of 84 accessions has been recently reported (Tomato Genome Sequencing Consortium et al., 2014). For important crops with no such databases, alternatives like ecotilling or amplicon sequencing can be considered for identifying natural loss-*S* alleles (Wang et al., 2012). These alleles could then be introgressed into susceptible lines. An alternative to breeding is:

### The Application of Genome Editing Strategies to Achieve Resistance

Once an *S* gene targeted by a particular TAL effector is identified, genome editing using tools such as TALENs and CRISPR-Cas9 can be employed to modify the EBE to prevent TAL effector binding. As mentioned before, this poses the risk of altering the endogenous regulation of the gene, so ideally several variants should be created and screened for any deleterious effects. The introduction of loss-of-function alleles to commercial, agronomically important varieties using these genome edition strategies can be expected to be subject to less restrictive regulation and greater public acceptance than GMOs (Araki and Ishii, 2015).

Alternatively, an artificial executor gene can be created by engineering the promoter of a classical *R* gene to have several EBEs corresponding to different TAL effectors. This approach has already been applied in rice and was shown to successfully confer resistance to the desired strains (Hummel et al., 2012; Zeng et al., 2015). A predicted, but not tested, alternative involves silencing of the *S* gene upon pathogen infection. For example an artificial microRNA can be designed directed specifically against the *S* host gene. However, to avoid the above-mentioned problem of affecting endogenous function, the expression of this microRNA should be under the control of a pathogen-inducible promoter. In this scenario, the EBE for a particular, prevalent TAL effector could be employed. The identification of naturally occurring and selected mutations presents a major advantage over *de novo* edited EBE knockouts since it avoids the risk of affecting endogenous *cis*-regulatory elements and thus plant gene functions. Yet, one should also raise the possibility that viability of such alleles might be genotype-dependent, they need therefore to be tested when moved to any elite recipient variety. In summary, if bacteria have evolved TAL effectors to take advantage of the presence of host genes to induce their expression for their benefit, plant breeders can counter with strategies directed at these TAL effectors to combat these plant pathogens.

## Conclusion

The discovery of the TAL effector-DNA binding code is greatly increasing our ability to identify new S proteins and better understand how diseases caused by *Xanthomonas* species occur in several major crops. Because of the highly conserved structure of TAL effectors and their mode of action to promote disease, standardized experimental pipelines can be developed in various *Xanthomonas*-host interactions to search for major *S* genes, provided that TAL effectors are at play. There is no doubt that in the near future, notably with the help of new sequencing technologies, extensive lists of new *S* genes will be generated to guide the search for pathogen-adapted loss-of-function alleles useful for marker-assisted breeding of loss-of-susceptibility. Less straightforward will be the challenge of finding such recessive resistance genes that cannot be easily broken, which will require better understanding of TAL effector diversity and evolution in pathogen populations.

## Conflict of Interest Statement

The authors declare that the research was conducted in the absence of any commercial or financial relationships that could be construed as a potential conflict of interest.

## Footnotes

1 http://www.mortalkombat.com/, Warner Bros. Interactive Entertainment.

2 http://dev.cassava-genome.cirad.fr/?q=snp
