# Corrigendum: MorTALKombat: the story of defense against TAL effectors through loss-of-susceptibility

**DOI:** 10.3389/fpls.2015.00647

**Published:** 2015-08-21

**Authors:** Mathilde Hutin, Alvaro L. Pérez-Quintero, Camilo Lopez, Boris Szurek

**Affiliations:** ^1^UMR IPME, Institut de Recherche Pour le Développement, IRD-CIRAD-Université Montpellier 2Montpellier, France; ^2^Biology Department, Universidad Nacional de ColombiaBogota, Colombia

**Keywords:** xanthomonas, plant disease susceptibility S genes, hubs, TAL effectors, agricultural biotechnology, loss-of-function alleles

There is an error in the statement about MeSWEET10a function in cassava bacterial blight. The TAL20-dependent activation of *MeSWEET10a* contributes to water soaking symptoms and also to bacterial growth in the plant, in contrast to what is reported in the review. The growth defect seen upon inoculation of Xam668Δ*TAL20* is small but it is statistically significant (Cohn et al., 2014). Accordingly, one should also read in Table 1 that TAL20 increases growth and water soaking (column “effect”).

## Conflict of interest statement

The authors declare that the research was conducted in the absence of any commercial or financial relationships that could be construed as a potential conflict of interest.
